# Olfactory Training and Oral Corticosteroid Therapy for Persistent Postinfectious Hyposmia *

**DOI:** 10.1055/s-0045-1802575

**Published:** 2025-09-19

**Authors:** Maria Victoria Bastos Tavares, Gabriel de Souza Mares, Maria Fernanda Danieluk, Maria Dantas Costa Lima Godoy, Renata Chade Aidar Balasso, Davi Ferreira Soares, Fábio Akira Suzuki

**Affiliations:** 1 Otorhinolaryngology and Cervico-Facial Surgery Service, Instituto de Assistência Médica ao Servidor Público Estadual de São Paulo (IAMSPE), São Paulo, SP, Brazil; 2 Department of Computer Science, CEDS, Instituto Tecnológico de Aeronáutica (ITA), São José dos Campos, SP, Brazil

**Keywords:** olfaction disorders, olfactory training, glucocorticoids

## Abstract

**Introduction:**

Postinfectious hyposmia gained special attention in the postpandemic era, and persistent cases are particularly difficult to treat. Many unproven therapies are used in clinical practice, including corticosteroids, with insufficient evidence.

**Objective:**

To establish the effectiveness of systemic corticosteroid therapy, associated with olfactory training, for persistent postinfectious hyposmia.

**Methods:**

Patients with persistent postinfectious hyposmia were divided, based on comorbidities, into control group (submitted to olfactory training alone) and test group (associated 7-day course of prednisone 40 mg). Olfactory evaluations were performed (visual analogue scale, Alcohol Sniff Test, and Connecticut Olfactory Test), at baseline, and at the 3- and 6-month follow-ups.

**Results:**

There was no statistically significant difference between the test (n = 10) and control (n = 7) groups ( *p* > 0.05) for primary outcomes (visual analogue scale, Alcohol Sniff Test, and Connecticut Olfactory Test), although there was statistically significant improvement of Alcohol Sniff Test scores in both groups at 6 months ( *p* > 0.05). The study's statistical power was reduced due to the small sample size. Even without randomization, the groups were not comparable only in terms of age ( *p* > 0.05). Although no statistically significant association was found, there was a clear tendency for improvement in the overall olfactory function, which may be spontaneous or due to olfactory training. No side effects were reported.

**Conclusion:**

There was no statistically significant benefit of systemic corticosteroid therapy for patients with persistent postinfectious hyposmia ( *p* > 0.05). Treatment with systemic corticosteroids should be individualized, and there is no consensus in the literature.

## Introduction

Olfaction is an important and often undervalued sensory system, contributing to identifying danger, increasing appetite, and influencing human emotion. 1 Its dysfunction is associated with increased risk of food poisoning, smoke inhalation, weight loss, anxiety, and depression. 1 Altered sense of smell is a common symptom in viral respiratory infections. In the post pandemic era, olfactory dysfunctions caught the attention of the scientific community, not only because of their especially high prevalence, but also because of its difficulty to treat. 2 3

In most cases of postinfectious smell dysfunction, recovery occurs in 2 to 3 weeks, in accordance with the regeneration time of the olfactory epithelium. 2 The mechanisms responsible for postinfectious hyposmia/anosmia include nasal obstruction and alteration of ciliary architecture, preventing the detection of odorants (localized conductive loss); injury to supporting cells or directly to olfactory neurons (sensory dysfunction), that can be caused by viral cytotoxic effect or by secondary immune injury; and injury to the olfactory bulb (central dysfunction). 4 Persistent postinfectious smell alteration may stem from: basal cell damage; viral persistence; or chronic inflammation with immune dysregulation and cell necrosis. 2 A variable immune response would explain why some patients have mild or no smell dysfunction and why others present persistent symptoms. 4 That is also the rationale behind corticosteroid use for treating postinfectious hyposmia. 5 However, there are no studies to date with proven benefit to recommend corticosteroid therapy 6 for postinfectious hyposmia.

The present study aims to establish the effectiveness of systemic corticosteroid therapy, associated with olfactory training, as a treatment for persistent postinfectious hyposmia, both of which are already widely used in otorhinolaryngological practice for the treatment of olfactory losses of various causes. 7 8 9

## Methods

A monocentric, controlled, non-randomized clinical trial was conducted, without blinding of researchers or patients. It was approved by the institutional Ethics in Research Committee (protocol number 50073721.7.0000.5463), and informed consent forms were signed by all participants.

Adult patients complaining of persistent olfactory alteration for at least 3 months after an upper respiratory infection, followed up at the *Hospital do Servidor Público Estadual* , from July 2021 to January 2023, were included. To this end, an active search was carried out for patients treated for upper respiratory infection with smell complaints, and 1,382 patients were then contacted regarding the persistence of hyposmia.

The exclusion criteria were patients with smell alterations prior to the infection; patients with nasofibrolaryngoscopy abnormalities that may justify olfactory loss from other causes, such as rhinitis; patients without hyposmia or anosmia in the diagnostic olfactory tests; patients without a confirmed upper respiratory infection; patients under 18 and over 70 years of age, who may present age-related smell alterations; and patients who refused to sign the informed consent form.

**Fig. 1:**
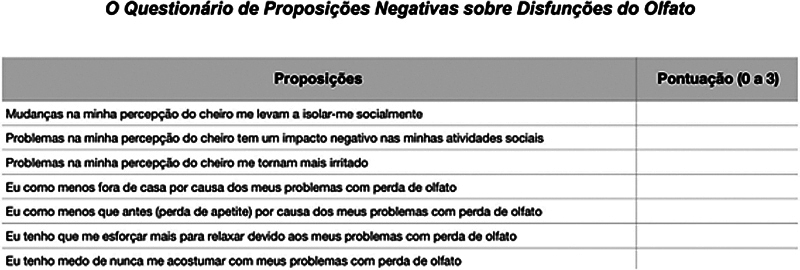
QOD-NS questions, validated for Brazilian Portuguese.

**Fig. 2:**
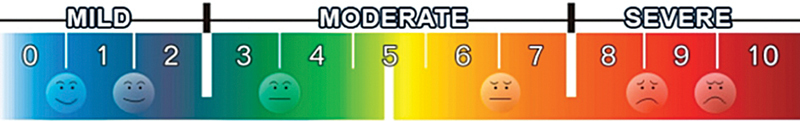
Visual analogue scale (VAS).

**Fig. 3:**
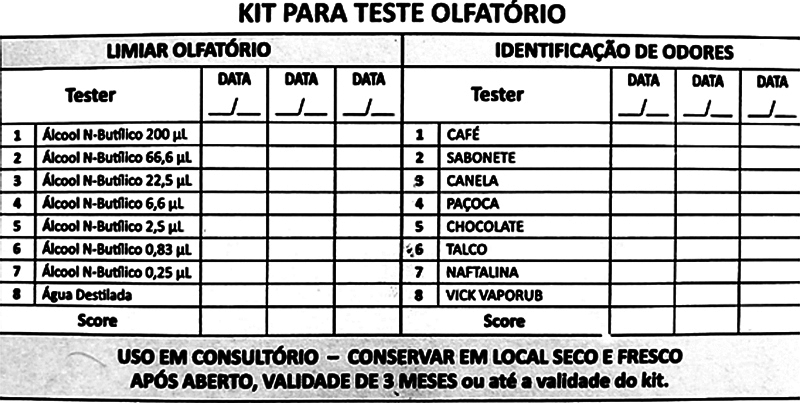
Connecticut Olfactory Test score sheet, validated for Brazilian Portuguese.

In the first evaluation, after clinical history and otorhinolaryngological physical examination, patients were submitted to: the Questionnaire of Olfactory Disorders-Negative Statements (QOD-NS) ( Figure 1 , Appendix 1 ), already well established in the literature;10 the visual analogue scale ( Figure 2 , Appendix 2 ); flexible nasofibrolaryngoscopy; the Alcohol Sniff Test ( Appendix 3 ) and the Connecticut Olfactory Test ( Figure 3 , Appendix 4 ), both of which are also well established in the literature. 11 12

Patients were divided into two groups based on comorbidities and/or contraindications to the use of systemic corticosteroids, such as diabetes and systemic arterial hypertension. The test group, composed of patients without comorbidities, was treated with prednisone 40 mg/day, orally, for 7 days, as established in previous studies, 13 in addition to classic olfactory training, 14 which consists of nasal instillation of 4 fragrances (lavender, eucalyptol/eucalyptus, citronellol/lemon, and eugenol/cloves) for 10 seconds each, 2 times a day, for 6 months. The control group, with patients with contraindications to corticosteroid therapy, underwent classical olfactory training alone.

Follow-up of the patients was carried out at 3 and 6 months, with evaluation of complaints, adherence to treatment and visual analogue scale, Alcohol Sniff Test, and Connecticut Olfactory Test scores.

## Results

Out of the 1,382 patients contacted, the study included a total of 19, 2 of whom dropped out, leaving 17 patients. The test group had 10 participants and the control group, 7.

Statistical analysis was performed using the R language v. 4.3.1 software (R Foundation for Statistical Computing, Vienna, Austria), parametric t-test for different samples, Friedman's and Mann-Whitney tests *.*

The baseline characteristics of the participants are shown in Table 1 and were generally well-balanced. The non-parametric t-test showed a statistically significant difference between groups ( *p* < 0.05) only in terms of age.

**Table 1:** Characteristics of participants

Variable	Test group		Control group		*p* -value
	Average	SD	Average	SD	
**Gender (M/F)**	4/6 (N)	40/60 (%)	3/4 (N)	43/57 (%)	
**Age (years)**	43.8	10.8	59.4	8.3	0.006
**QOD-NS (points)**	8	4.7	5.5	7.3	0.3975
**Hyposmia time (months)**	14	6.6	13.7	6.5	0.9326

**Abbreviations** : SD, standard deviation; M, male; F, female; QOD-NS, Questionnaire of Olfactory Disorders-Negative Statements.

**Graph 1:**
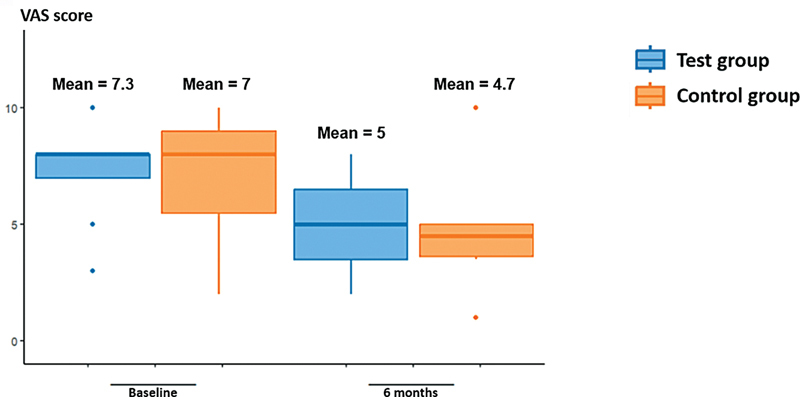
Visual analogue scale (VAS) results.

The results at baseline, and at 3 and 6 months are described in Table 2 and Graphs 1 2 3 . There was no statistically significant difference between groups ( *p* > 0.05), according to the non-parametric T-test, for the primary outcome of the variables of interest (visual analogue scale, Alcohol Sniff Test, and Connecticut Olfactory Test).

**Table 2:** Variables of interest for primary outcome (Visual Analogue Scale, Alcohol Sniff Test and Connecticut Olfactory Test), at baseline, and at the 3- and 6-month follow-ups, comparing test and control groups

Time	Variable	Test group		Control group		*p* -value
		Average	SD	Average	SD	
**Baseline**	VAS (points)	7.3	2.2	7	2.8	0.7958
	Alcohol Sniff Test (cm)	15.5	8.4	9.7	12.4	0.2695
	Connecticut (points)	3	1.7	2.3	0.8	0.4012
**3 months**	VAS (points)	4.4	2.1	6	−	0.4978
	Alcohol Sniff Test (cm)	20.6	6.1	9.7	−	0.1452
	Connecticut (points)	3.5	2.2	4.5	−	0.7075
**6 months**	VAS (points)	5	2.3	4.7	2.7	0.6254
	Alcohol Sniff Test (cm)	18.8	6.2	13.5	9.3	0.1514
	Connecticut (points)	4.4	1.2	3.3	1	0.7004

**Abbreviations** : SD, standard deviation; VAS, visual analogue scale; Connecticut, Connecticut Olfactory Test.

**Graph 2:**
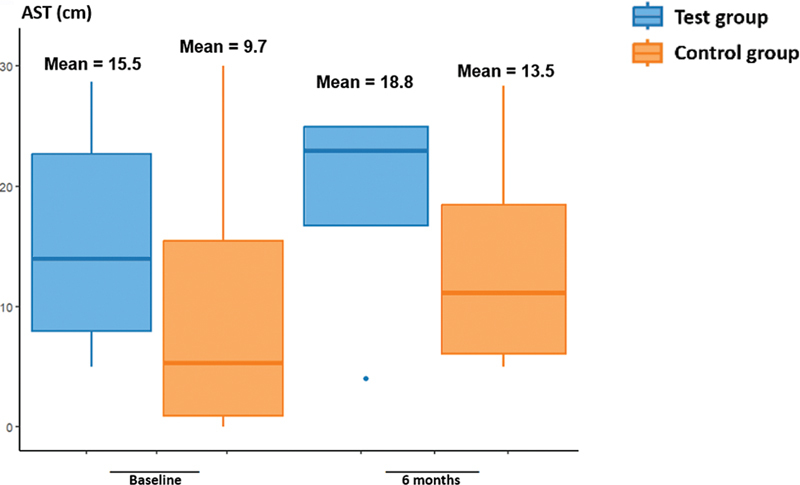
Alcohol Sniff Test (AST) results.

**Graph 3:**
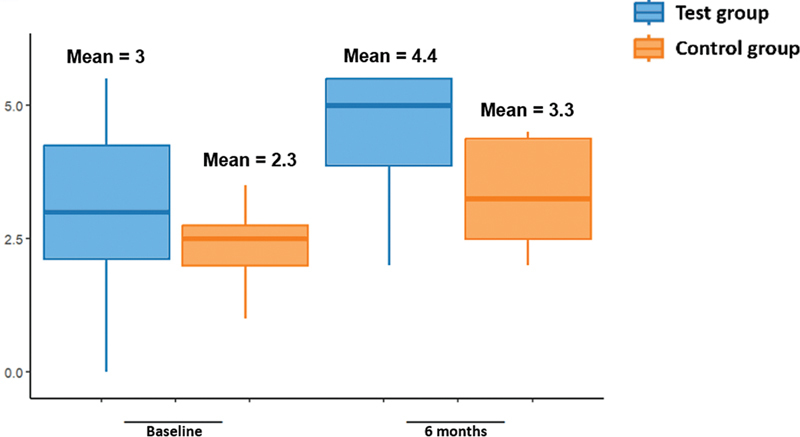
Connecticut Olfactory Test results.

The improvement of scores over time was assessed by the Friedman's test, which showed statistical significance ( *p* = 0.0231) for Alcohol Sniff Test results in same-group analysis.

**Graph 4:**
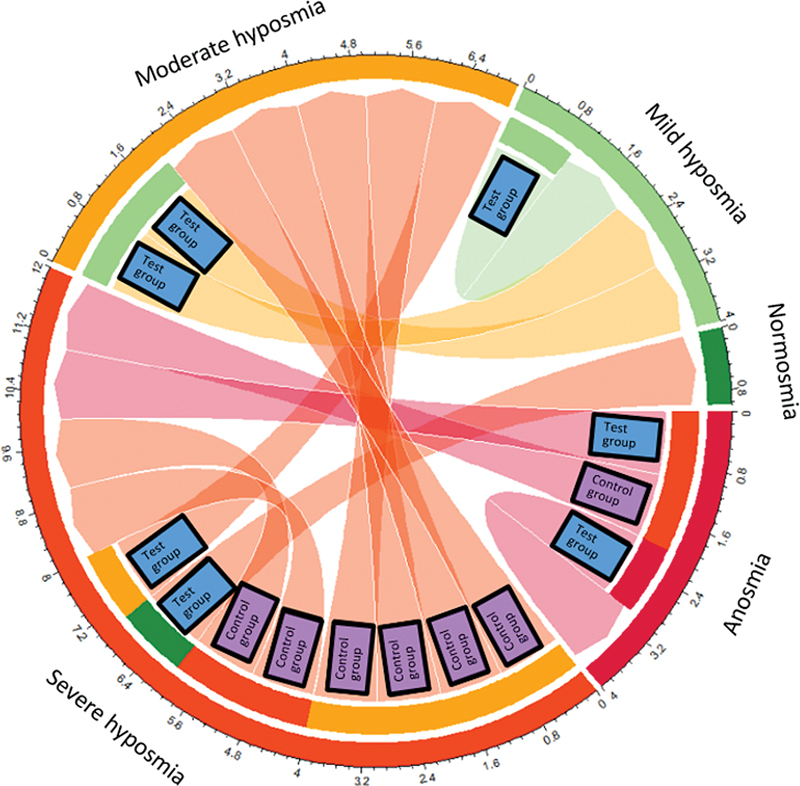
Chord chart of Connecticut Olfactory Test category, from baseline to 6-month follow-up, all participants.

For categorical variables, the Mann-Whitney test was calculated, and there was no statistically significant difference between groups ( *p* > 0.05), in accordance with the numerical evaluation. There was an improvement in the category of smell alteration in 71.5% of patients, and maintenance of the category in 28.5% of patients ( Graph 4 ).

No complications associated with therapy were reported.

## Discussion

There was no statistically significant difference to affirm benefit from systemic corticosteroid therapy in persistent postinfectious smell dysfunction ( *p* > 0.05). Improvement was observed in both groups, which suggests that olfactory training may be a relevant treatment option.

A search of the literature has shown a few similar articles. Le Bon et al., 15 in a study with 27 patients, found benefit for use of oral corticosteroids in post-coronavirus disease 2019 (COVID-19) hyposmia ( *p* = 0.007), but the olfactory alterations were acute, only 5 weeks after infection. Pendolino et al., 16 in a 6-month cohort of 44 patients with prolonged hyposmia after COVID-19, did not find superiority of corticosteroids compared to olfactory training. Genetzaki et al., 5 in a non-randomized trial with 131 participants with non-COVID postinfectious hyposmia, also found no difference between isolated olfactory training and a combination of that with corticosteroids, but the duration of olfactory loss was variable and only described for subgroups of those who used corticosteroids (mean 6.85 ± 1.8 months in improved patients with inflammatory background versus 2.85 ± 1.2 months in improved patients without inflammatory background), suggesting a shorter duration of dysfunction than in our study.

According to the international consensus on olfaction, published in 2022, 6 there is a lack of evidence for the use of corticosteroids in the treatment of smell disorders unrelated to chronic sinusitis or allergic rhinitis. In the absence of these conditions, there are few data to recommend the use of oral corticosteroids.

Olfactory training, first described by Hummel et al., in 2009, 7 is recommended for all types of smell loss, including posttraumatic, postinfectious, idiopathic, and age- or Parkinson's disease-related, and the benefit seems to be greater in postinfectious smell loss. It is a treatment that has no side effects, has proven its safety, and is easy to perform. However, adherence to treatment is a challenge.

The evaluation of the patients' adherence to olfactory training was performed subjectively over follow-up visits. No breaks in adherence were reported for more than 1 month, usually justified by the lack of perception of improvement. Patients were willing to undergo prolonged treatment after adequate orientation. At the end of the trial, after delivery of objective results, patients expressed satisfaction with scores improvement, which shows how useful objective scores in clinical practice can be, offering evidence of treatment response, given its gradual and prolonged nature, when patient perception is diminished.

Within each group, there was a clear trend of improvement in olfactory function. For disorders with a high rate of spontaneous improvement, demonstrating the benefit of possible treatments is difficult, since the cause of the improvement may be the treatment applied or the natural evolution of the disease itself. However, it would not be ethical to include a control group without any treatment to evaluate improvement over time, in view of the proven benefit of olfactory training for olfactory dysfunction. However, only 3 patients in the test group and 2 patients in the control group had less than 1 year of olfactory loss (respectively 9, 7, and 6 months in the test group and 3 and 9 months in the control group), which reduces the chance of spontaneous improvement, overall, in the study.

The statistical power of the study was reduced by a few factors. Sample size did not achieve the estimated 26 patients in each group for statistical significance. The two dropouts reported improved olfactory function as a justification for leaving the study.

The groups were not randomized, which could account for a selection bias. However, the current study does not aim to define the theoretical efficacy or safety of systemic corticosteroid therapy. The criteria for dividing the groups are compatible with daily clinical life, considering the profile of comorbidities and the risk-benefit assessment in selecting therapies. There were no reports of side effects from our short-term use of corticosteroids. Even without randomization, the groups were not comparable only in terms of age ( *p* < 0.05). This difference, in addition to sample size, may have contributed to the lack of statistical significance between groups.

## Conclusion

There was no statistically significant difference to affirm that the use of systemic corticosteroid therapy for patients with persistent smell alteration after an upper airway infection is beneficial ( *p* > 0.05). The choice of treatment with systemic corticosteroids should be individualized, and there is still no consensus in the literature.

Olfactory training remains a therapeutical choice for management of postinfectious smell dysfunction.

## Notes

Appendix 1 - Questionnaire of Negative Propositions on Smell Disorders (QOD-NS)
The Questionnaire of Olfactory Disorders-Negative Statements (QOD-NS) is an important instrument used to assess the impact on quality of life of patients with smell alterations, with a good correlation with objective smell tests and good psychometric validity. 16 The questions are shown in Figure 1 below.

## Notes

Appendix 2 – Analogue Visual Scale (AVS)
Patients were shown the following visual analogue scale ( Fig. 2 ) and asked how altered their olfaction were.

## Notes

Appendix 3 - Alcohol Sniff Test
Described in 1997 by Davidson et al. 11 , the test measures the distance in centimeters (cm) at which the patient perceives the odor of 70% alcohol. A 30-cm ruler is used, positioned so that the 0-cm mark by the patient's nostril. A 70% alcohol swab is placed on the other end of the ruler. With each inhalation of the patient, the swab is brought closer by 1 cm. The distance at which the patient signals smelling the alcohol swab is noted. Three measurements are taken, and the result is the mean.
Anosmia is considered when the mean is lower than or equal to 5 cm; normosmia, over 12 cm; and hyposmia, less than or equal to 12 cm and more than 5 cm.

## Notes

Appendix 4 - Connecticut Olfactory Test
Validated for Brazil in 2020 by Phenolio et al. 12 , the Connecticut Olfactory Test is a tool for assessing olfactory function consisting of two parts: olfactory threshold and odor discrimination ( Fig. 3 ).

The threshold is measured by presenting two vials near the patient's nostrils, one of which is always distilled water, and the other is N-butyl alcohol in increasing concentrations. Each presentation is performed twice, alternating vials. The threshold is the concentration of N-butyl alcohol at which the patient correctly does not identify the odor of distilled water and identifies the odor of alcohol. If the patient does not identify N-butyl alcohol in the concentration presented on either of the two occasions, or if the patient identifies the odor in the distilled water on either of the two occasions, this concentration not the threshold and the examination is carried on with a new presentation of distilled water and a more concentrated N-butyl alcohol. The score is calculated according to the number of the vial identified as the threshold, with 0 points if any concentration is identified and 7 points if the lowest N-butyl alcohol concentration is identified ( Fig. 3 ).
Discrimination is tested with vials containing eight different odorants. The patient is unaware of the contents of each vial. A list is given to the patient, containing the odorants in the test plus eight odorants absent from the test. The patient is instructed to choose which odorant from this list can be smelled in the vial. The last odorant is a control test of trigeminal function, containing Vick Vaporub, the only odorant not present in the list and which is not scored. The vial can be presented as many times as the patient requests. Score from 0 to 7 how many vials were correctly identified. The total score is the average of the threshold and discrimination points. It is classified as normosmia from 6 to 7 points, mild hyposmia from 5 to 5.75, moderate hyposmia from 4 to 4.75, severe hyposmia from 2 to 3.75, and anosmia from 0 to 1.75.
