# Supplementary material for: Olfactory Training and Oral Corticosteroid Therapy for Persistent Postinfectious Hyposmia
Source: Int Arch Otorhinolaryngol. 2025 Sep 19;29(3):1–7. doi: 10.1055/s-0045-1802575 (PMC12449106; doi:10.1055/s-0045-1802575)
Supplement: Supplementary file 1 — Supplementary Appendix [file 10-1055-s-0045-1802575-s241791.pdf]

Appendix 1 - Questionnaire of Negative Propositions on Smell Disorders (QOD-NS)

The Questionnaire of Olfactory Disorders-Negative Statements (QOD-NS) is an important instrument used to assess the impact on quality of life of patients with smell alterations, with a good correlation with objective smell tests and good psychometric validity.[16] The questions are shown in ►**Figure 1** below.

Appendix 2 – Analogue Visual Scale (AVS)

Patients were shown the following visual analogue scale (►**Fig. 2**) and asked how altered their olfaction were.

Appendix 3 - Alcohol Sniff Test

Described in 1997 by Davidson et al.[11], the test measures the distance in centimeters (cm) at which the patient perceives the odor of 70% alcohol. A 30-cm ruler is used, positioned so that the 0-cm mark by the patient's nostril. A 70% alcohol swab is placed on the other end of the ruler. With each inhalation of the patient, the swab is brought closer by 1 cm. The distance at which the patient signals smelling the alcohol swab is noted. Three measurements are taken, and the result is the mean.

Anosmia is considered when the mean is lower than or equal to 5 cm; normosmia, over 12 cm; and hyposmia, less than or equal to 12 cm and more than 5 cm.

Appendix 4 - Connecticut Olfactory Test

Validated for Brazil in 2020 by Phenolio et al.[12], the Connecticut Olfactory Test is a tool for assessing olfactory function consisting of two parts: olfactory threshold and odor discrimination (►**Fig. 3**).

The threshold is measured by presenting two vials near the patient's nostrils, one of which is always distilled water, and the other is N-butyl alcohol in increasing concentrations. Each presentation is performed twice, alternating vials. The threshold is the concentration of N-butyl alcohol at which the patient correctly does not identify the odor of distilled water and identifies the odor of alcohol. If the patient does not identify N-butyl alcohol in the concentration presented on either of the two occasions, or if the patient identifies the odor in the distilled water on either of the two occasions, this concentration not the threshold and the examination is carried on with a new presentation of distilled water and a more concentrated N-butyl alcohol. The score is calculated according to the number of the vial identified as the threshold, with 0 points if any concentration is identified and 7 points if the lowest N-butyl alcohol concentration is identified (►**Fig. 3**).

Discrimination is tested with vials containing eight different odorants. The patient is unaware of the contents of each vial. A list is given to the patient, containing the odorants in the test plus eight odorants absent from the test. The patient is instructed to choose which odorant from this list can be smelled in the vial. The last odorant is a control test of trigeminal function, containing Vick Vaporub, the only odorant not present in the list and which is not scored. The vial can be presented as many times as the patient requests. Score from 0 to 7 how many vials were correctly identified.

The total score is the average of the threshold and discrimination points. It is classified as normosmia from 6 to 7 points, mild hyposmia from 5 to 5.75, moderate hyposmia from 4 to 4.75, severe hyposmia from 2 to 3.75, and anosmia from 0 to 1.75.
